# Association of the *TP53* codon 72 polymorphism and breast cancer risk: a meta-analysis

**DOI:** 10.1186/2193-1801-3-749

**Published:** 2014-12-17

**Authors:** Meire Luzia Gonçalves, Sarah Moreira Borja, Jacqueline Andréia Bernardes Leão Cordeiro, Vera Aparecida Saddi, Flávio Monteiro Ayres, Cesar Augusto Sam Tiago Vilanova-Costa, Antonio Márcio Teodoro Cordeiro Silva

**Affiliations:** Departamento de Medicina, Pontifícia Universidade Católica de Goiás, Av. Universitária 1.069, Setor Universitário, Goiânia, Goiás CEP 74.605-010 Brazil; Faculdade de Enfermagem, Universidade Federal de Goiás, Goiânia, Goiás CEP 74605-080 Brazil; Laboratório de Oncogenética e Radiobiologia, Hospital Araújo Jorge, Associação de Combate ao Câncer em Goiás, Goiânia, Goiás CEP 74605-070 Brazil; Unidade Universitária de Ciências Exatas e Tecnológicas, Universidade Estadual de Goiás, Anápolis, Goiás CEP 75132-400 Brazil; Programa de Pós-Graduação Stricto Sensu em Ciências Ambientais e Saúde, Pontifícia Universidade Católica de Goiás, Goiânia, Goiás CEP 74065-140 Brazil

**Keywords:** Arg72Pro, Genetic susceptibility, p53, R72P, Single nucleotide polymorphism, Breast cancer

## Abstract

This study was conducted in order to investigate the implications of the R72P polymorphism in the *TP53* gene in breast cancer risk. The enlightenment of this matter might provide a piece of information about the potential implications of this polymorphism in patient risk. A meta-analysis was conducted considering a large sample size from studies with conflicting results on the R72P polymorphism in breast cancer patients. Relevant studies were selected from PubMed and SciELO databases for data extraction and statistical analysis. Database was built according to the continent and considering the genotype frequencies, sample size and genotyping methodology. The dominant models (RR vs RP + PP and RR + RP vs. PP), homozygous (RR vs. PP), heterozygous (RR vs. RP and RP vs. PP) and the allele (R vs. P) were used. Genotype frequencies were summarized and evaluated by χ^2^ test of heterogeneity in 2×2 contingency tables with 95% CIs. Odds Ratios (OR) were calculated with a fixed-effect model (Mantel-Haenszel) or a random-effect model (DerSimonian-Laird) if the studies were considered homogeneous (P > 0.05) or heterogeneous (P < 0.05), respectively, using BioEstat® 5.0 software. Supported by a large sample size composed by 25,629 cases and 26,633 controls from 41 studies, we found significant association between the R72P polymorphism in the *TP53* gene and the breast cancer risk. The overall data shows an increased risk due to the P allele dominant model, but not in Asia where the risk was associated with the R allele and R dominant model.

## Introduction

The R72P polymorphism in the TP53 gene results of the transversion G → C in the second position of the codon 72 at exon 4. Both the polymorphic alleles vary among ethnic groups (Dokianakis et al. 2000) and geographic location, where the P allele is more frequent toward the equador line purportedly as a protective factor against UV rays (Damin et al. 2006; Olivier et al. 2002). P53 variant proteins have an arginine (R) or a proline (P) encoded by codon 72, which differ in structure and function, specially concerning cell cycle progress (Chang-Claude et al. 2009; Schmidt et al. 2009; Thomas et al. 1999; Petitjean et al. 2007; Dumont et al. 2003).

Breast cancer is an heterogeneous sporadic or hereditary disease (Lima et al. 2006). The hereditary syndrome affects 10% of patients, of which 5% has high penetrance mutations in genes like *BRAC1* and *BRCA2* (BRCA1/2) (Pinto et al. 2007). *BRCA1/2* and *TP53* are susceptibility genes that confer high-risk of breast cancer (Oluwagbemiga et al. 2012). Evidences that the R72P polymorphisms in the *TP53* gene can differently promote the transcription of *BRAC1/2* have widely supported studies on R72P role in breast tumorigenesis (Sinilnikova et al. 2009; Lum et al. 2008; Osorio et al. 2008; Gochhait et al. 2007; Cavallone et al. 2008; Baynes et al. 2007; Tommiska et al. 2005; Martin et al. 2003; Huang et al. 2003), *e.g.*, (1) the P variant binds greater to transcriptional machinery (Thomas et al. 1999) and thus shows higher rates of G1 arrest than the R variant protein (Petitjean et al. 2007; Gochhait et al. 2007); (2) the decreased efficiency of the P variant at triggering apoptosis (Chang-Claude et al. 2009; Dumont et al. 2003), mainly due to its decreased ubiquination by MDM2 (Sinilnikova et al. 2009; Lum et al. 2008; Gochhait et al. 2007; Francisco et al. 2010) and to its increased efficiency to bind the inhibitor of apoptosis-stimulating protein of p53 (iASPP) (Schmidt et al. 2009; Bergamaschi et al. 2006).

In the present study, a meta-analysis was conducted considering a large sample size from studies with conflicting results on the R72P polymorphism in breast cancer patients. The enlightenment of this matter might provide a piece of information about the potential implications of this polymorphism in patient’s risk.

## Material and methods

### Identification and eligibility of relevant studies

A literature search was conducted in SciELO (Scientific Eletronic Library Online) and PubMed databases by using the keywords: *p53*, *polymorphism*, *breast cancer*. Additional studies were searched among the references surveyed in the databases. Eligible studies were selected regardless of sample size, but had to meet the following criteria of inclusion: (a) the studies were published from 2002 to 2012; (b) the association between the R72P polymorphism and breast cancer were investigated; (c) the studies were case–control design; (d) genotyping was carried out by molecular biology methods, such as PCR, RFLP-PCR and DNA sequencing; (e) the reference was published in English; (f) histological confirmation of breast cancer diagnosis was performed; and (g) the genotype distributions were available for estimating odds ratios (OR) and 95% confidence intervals (CI).

### Data extraction

Two investigators independently extracted data and reached a consensus on all of the items. A third investigator took part of data extraction in case of disagreement in any of the items. The data extracted regarded country of origin, first author, and year of publication, number of cases and controls, and genotype frequencies.

### Statistical analysis

In the current meta-analysis, the dominant models (RR *vs* RP + PP and RR + RP *vs.* PP), homozygous (RR *vs.* PP), heterozygous (RR *vs.* RP and RP *vs.* PP) and the allele (R *vs.* P) were used. Genotype frequencies were summarized and evaluated by χ^2^ test of heterogeneity in 2×2 contingency tables with 95% CIs (Böhning et al. 2002). Odds Ratios (OR) were calculated with a fixed-effect model (Mantel-Haenszel) or a random-effect model (DerSimonian-Laird) if the studies were considered homogeneous (*P* > 0.05) or heterogeneous (*P* < 0.05), respectively. The OR and their corresponding 95% CI were used to test the association between the 72 codon polymorphism and breast cancer. All analyses were performed with BioEstat® 5.0 software. To estimate a combined effect, OR were calculated for both fixed and random effect analyses, by applying 95% CIs and individual or combined weights for the studies (Li et al. 2012; Conn et al. 2012; Manning et al. 2011; Higgins et al. 2008).

## Results and discussion

### Study inclusion and characteristics

**Figure 1:**
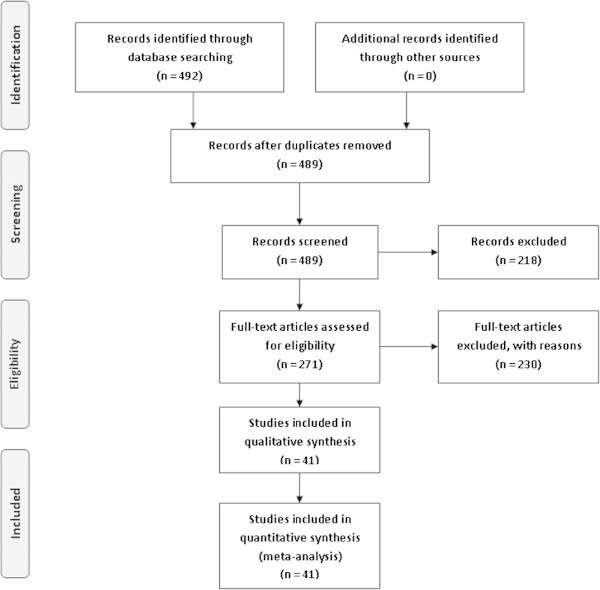
**Flow diagram of the studies evaluated for meta-analysis.**

A total of 492 studies were screened, of which three were found in both PubMed and SciELO. From the 489 studies screened, 218 were published before 2002 and 20 were reviews or published in another language than English, or both. The remaining 271 records were assessed for eligibility and 41 fulfilled the criteria of inclusion (Figure 1). From the selected studies, a database was built considering the continent, genotype frequencies, sample size and genotype methodology. All together, the 41 studies that met the inclusion criteria and were identified as eligible article, yielding 25,629 cases and 26,633 controls.

In the last 10 years, eligible studies on R72P polymorphism in the *TP53* gene in breast cancer were mostly from Europe with 19 articles, followed by Asia, America and Africa with 14, 6 and 2, respectively. Subject’s age was collected, when available, showing that mean age of patients was 51.9 y.o. and of control subjects 48.1 y.o. The genotyping for p53 codon 72 polymorphism was performed using Polymerase Chain Reaction (PCR), Allele-Specific PCR (AS-PCR), Amplifluor®, GoldenGate® Genotyping Assay (GGA), PCR-Denaturing Gradient Gel Electrophoresis (PCR-DGGE), PCR-Restriction Fragment Length Polymorphism (PCR-RFLP), sequencing and Taqman PCR. Breast cancer patients and controls subjects were mainly heterozygous in Asia (50.1%; 48.0%) and Africa (43.9%; 49.7%), while the RR homozygous was predominant in America (53.6%; 54.5%) and Europe (54.1%; 53.4%). The R allele was predominant in breast cancer patients from America and Europe (73.3%, each), Africa (63.2%) and Asia (58.3%).

### Quantitative synthesis

**Figure 2:**
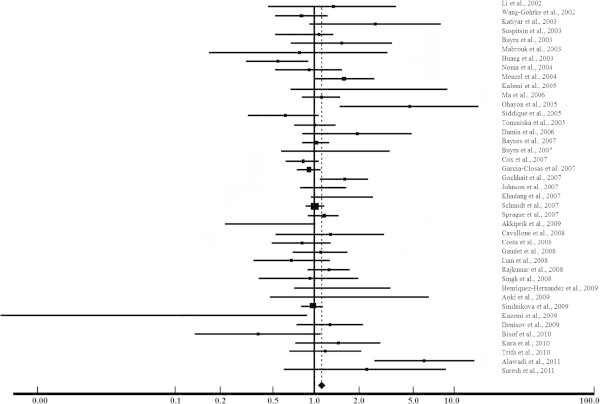
**Meta-analysis evaluation of the dominant model RR + RP** ***vs.*** **PP.**

**Figure 3:**
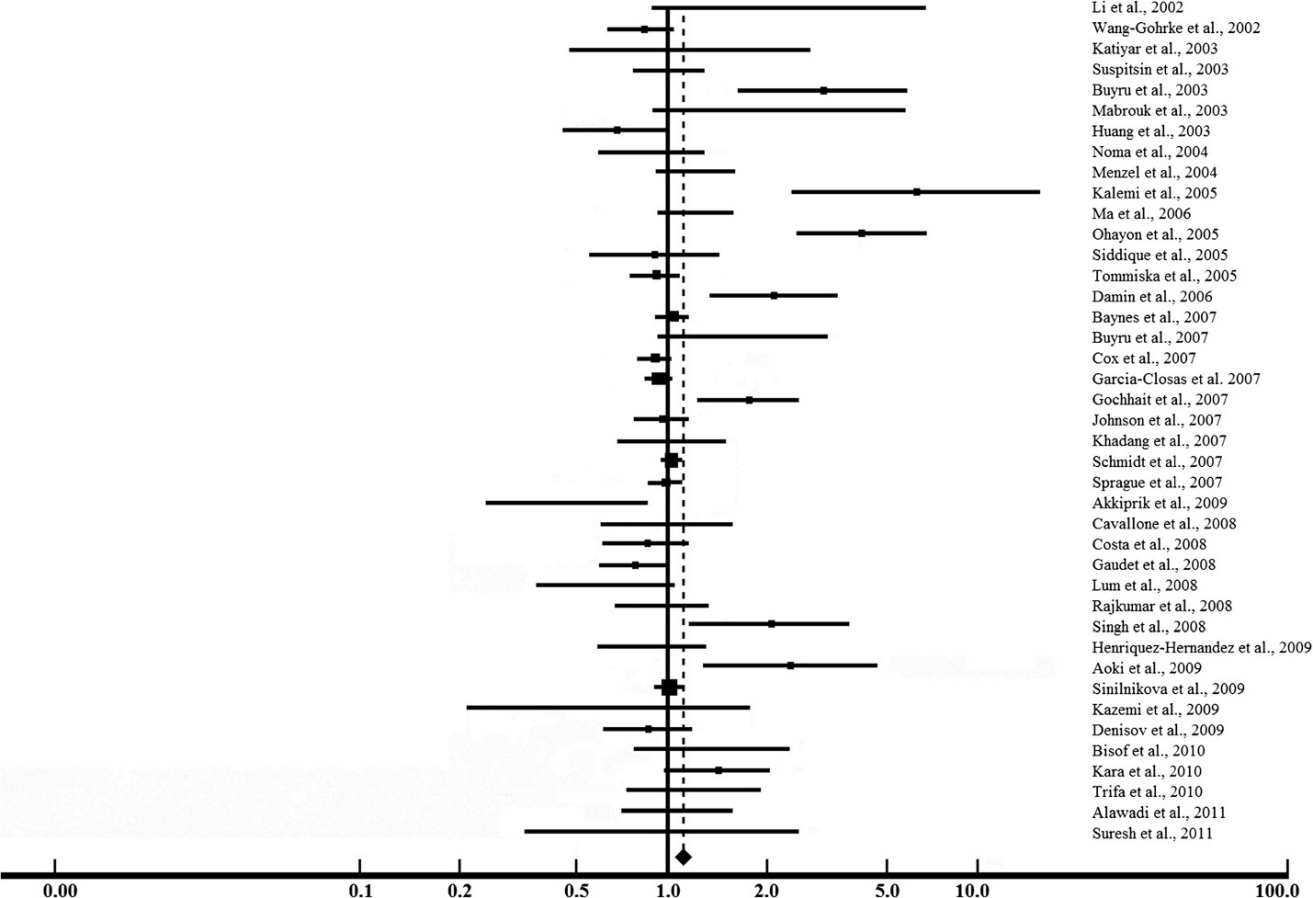
**Meta-analysis evaluation of the dominant model RR** ***vs.*** **RP + PP.**

The dominant models RR + RP *vs.* PP and RR *vs.* RP + PP had OR calculated using a random-effect model. No association between breast cancer risk and the dominant model RR + RP *vs.* PP (OR = 1.09; 95% CI 0.98-1.22) was found (Figure 2 and Table 1). By the other hand, our findings for RR *vs.* RP + PP (OR = 1.11; 95% CI 1.02-1.21) showed a markedly increased risk of breast cancer associated with the RP and PP genotypes, considering the P allele as dominant (Figure 3 and Table 1). In agreement with our results, the PP genotype was previously associated with higher risk for breast cancer (Huang et al. 2003; Rajkumar et al. 2008). Among unselected breast cancer patients, the PP genotype also predicted poor survival and a 2-fold increased risk of death (Tommiska et al. 2005).

**Table 1:** **Meta-analysis of the R72P polymorphism of the gene** ***TP53*** **on breast cancer**

Studies/continent	No. of case/control	RR + RP ***vs.*** PP	RR ***vs.*** RP + PP	R ***vs.*** P
		OR (95% CI)	OR (95% CI)	OR (95% CI)
**Africa**	**Σ=189/181**	1.13 (0.64-2.01)	1.40 (0.91-2.15)	1.16 (0.86-1.56)
Trifa et al., 2010	159/132	1.19 (0.65-2.19)	1.21 (0.75-1.96)	1.15 (0.83-1.61)
Mabrouk et al., 2003	30/49	0.78 (0.18-3.40)	2.32 (0.93-5.78)	1.59 (0.78-3.27)
**America**	**Σ=6,483/8,011**	0.99 (0.87-1.12)	1.05 (0.88-1.26)	0.98 (0.92-1.02)
Damin et al., 2006	118/202	2.05 (0.83-5.09)	2.22 (1.40-3.53)	1.79 (1.25-2.55)
Aoki et al., 2009	72/90	1.78 (0.48-6.60)	2.47 (1.31-4.66)	1.85 (1.14-3.00)
Gaudet et al., 2008	578/390	1.11 (0.70-1.76)	0.78 (0.61-1.01)	0.88 (0.71-1.08)
Cox et al., 2007	1,477/2,224	0.83 (0.63-1.08)	0.92 (0.81-1.05)	0.92 (0.83-1.02)
Garcia-Closas et al. 2007	2,585/3,251	0.92 (0.75-1.12)	0.94 (0.84-1.04)	0.95 (0.87-1.03)
Sprague et al., 2007	1,653/1,854	1.16 (0.89-1.52)	1.00 (0.87-1.14)	1.02 (0.92-1.14)
**Asia**	**Σ=2,570/2,833**	1.21 (0.89-1.64)	1.08 (0.89-1.32)	*1.09 (1.01-1.17)
Lum et al., 2008	393/80	0.69 (0.37-1.30)	0.64 (0.39-1.06)	0.73 (0.51-1.03)
Gochhait et al., 2007	243/333	1.66 (1.12-2.46)	1.85 (1.28-2.67)	1.56 (1.23-1.92)
Huang et al., 2003	200/282	0.54 (0.32-0.92)	0.70 (0.48-1.02)	0.72 (0.55-0.93)
Ma et al. 2006	404/472	1.14 (0.82-1.59)	1.25 (0.95-1.66)	1.16 (0.96-1.40)
Rajkumar et al., 2008	250/500	1.27 (0.89-1. 80)	0.97 (0.69-1.37)	1.08 (0.87-1.34)
Li et al., 2002	28/50	1.38 (0.48-3.99)	2.54 (0.93-6.94)	1.68 (0.87-3.27)
Alawadi et al., 2011	288/188	6.12 (2.66-14.08)	1.08 (0.71-1.63)	1.31 (1.00-1.71)
Suresh et al., 2011	35/37	2.28 (0.59-8.91)	0.95 (0.35-2.57)	1.21 (0.62-2.34)
Kazemi et al., 2009	42/57	0.05 (0.003-0.89)	0.65 (0.23-1.84)	0.65 (0.37-1.15)
Singh et al., 2008	104/105	0.91 (0.40-2.06)	2.26 (1.21-3.85)	1.42 (0.96-2.11)
Khadang et al., 2007	221/205	1.60 (0.95-2.68)	1.04 (0.70-1.54)	1.17 (0.89-1.54)
Siddique et al., 2005	94/265	0.62 (0.34-1.12)	0.92 (0.57-1.49)	0.83 (0.59-1.16)
Noma et al., 2004	191/218	0.93 (0.54-1.60)	0.92 (0.62-1.35)	0.82 (0.61-1.10)
Katiyar et al., 2003	77/41	2.79 (0.93-8.39)	1.22 (0.51-2.95)	1.38 (0.80-2.36)
**Europe**	**Σ=16,387/15,608**	1.03 (0.95-11.25)	1.13 (1.00-1.27)	1.02 (0.99-1.06)
Sinilnikova et al., 2009	3,959/3,052	0.97 (0.80-1.16)	1.01 (0.92-1.11)	1.00 (0.93-1.08)
Cavallone et al., 2008	157/112	1.29 (0.52-3.21)	1.00 (0.62-1.63)	1.04 (0.71-1.53)
Baynes et al., 2007	2,023/2,197	1.04 (0.82-1.30)	1.05 (0.93-1.18)	1.04 (0.95-1.14)
Tommiska et al., 2005	1,551/733	1.02 (0.72-1.43)	0.93 (0.78-1.11)	0.96 (0.83-1.10)
Akkiprik et al. 2009	95/108	0.39 (0.14-1.12)	1.38 (0.78-2.42)	1.02 (0.65-1.91)
Kara et al., 2010	204/192	1.48 (0.73-3.00)	1.45 (0.98-2.15)	1.34 (0.99-1.81)
Bisof et al., 2010	95/107	0.48 (0.22-1.04)	0.48 (0.27-0.87)	0.58 (0.39-0.86)
Denisov et al., 2009	297/275	1.28 (0.73-2.23)	0.87 (0.62-1.20)	0.96 (0.75-1.25)
Henrıquez-Hernandez et al. 2009	135/295	1.60 (0.72-3.54)	0.90 (0.60-1.36)	1.03 (0.74-1.43)
Costa et al., 2008	248/646	0.81 (0.49-1.32)	0.86 (0.64-1.16)	0.87 (0.69-1.10)
Buyru et al., 2007	115/63	1.44 (0.58-3.57)	1.77 (0.96-3.29)	1.52 (0.96-2.43)
Johnson et al., 2007	472/2,462	1.17 (0.79-1.74)	0.98 (0.80-1.19)	1.01 (0.87-1.19)
Schmidt et al. 2007	5,191/3,834	1.01 (0.86-1.18)	1.05 (0.96-1.14)	1.03 (0.96-1.10)
Kalemi et al., 2005	42/51	2.52 (0.69-9.26)	6.35 (2.54-15.84)	3.29 (1.73-6.25)
Ohayon et al., 2005	132/167	4.86 (1.52-15.53)	4.29 (4.68-6.96)	3.10 (2.10-4.56)
Menzel et al., 2004	475/302	1.64 (0.97-2.76)	1.25 (0.94-1.67)	1.27 (1.01-1.60)
Buyru et al., 2003	115/76	1.61 (0.69-3.73)	3.23 (1.7-6.00)	2.09 (1.36-3.22)
Suspitsin et al., 2003	529/393	0.86 (0.52-1.41)	1.04 (0.80-1.35)	1.00 (0.81-1.23)
Wang-Gohrke et al., 2002	552/543	0.82 (0.53-1.26)	0.85 (0.67-1.07)	0.87 (0.72-1.05)
**Overall**	25,629/26,633	1.09 (0.98-1.22)	*1.11 (1.02-1.21)	1.02 (1.00-1.05)
**Heterogeneity**		χ^2^ = 80.19	χ^2^ = 146.02	χ^2^ = 6.54
		*P* = 0.0002	*P<0.0001*	*P* = 0.088

The P allele has been associated with earlier breast cancer onset in *BRCA1/2* mutation carriers (Tommiska et al. 2005; Martin et al. 2003), probably due to penetrance modification in *BRCA1* (Martin et al. 2003) and to the lower ability of the P variant to induce apoptosis in genotoxic stress (Chang-Claude et al. 2009; Dumont et al. 2003). However, most of the studies selected for this meta-analysis have failed to detect any implication of R72P to breast cancer risk. Among most of the selected references, no risk modification by R72P was found in wild type *BRCA1/2* and mutation carriers, even if the age of diagnosis or tumor stage were regarded (Sinilnikova et al. 2009; Lum et al. 2008; Cavallone et al. 2008; Baynes et al. 2007; Tommiska et al. 2005). Our overall data showed an association of risk increase with PP genotype, but not with the alleles alone (R *vs.* P; OR = 1.02; 95% CI 1.00-1.05), as described in Table 1. The lack of implication concerning P allele alone might be explained by the R allele in the heterozygous, because the R variant may act in a codominant mode to decrease breast cancer risk and to detain the onset in sporadic cases (Lum et al. 2008). Although our overall data show no association with the alleles alone, the analysis of 2,570 cases and 2,833 controls from Asia demonstrated a markedly increase of the R allele frequency in breast cancer patients (R vs. P; OR = 1.09; 95% CI 1.01-1.17), as detailed in Table 1.

Ethnic and geographical nonspecific factors, further to allele frequencies variations in different health populations, have been argued as the reason to the controversial data on R72P role in breast cancer (Dokianakis et al. 2000; Lum et al. 2008; Huang et al. 2003). Worth of note in this regard is that, in our meta-analysis, R allele was the most frequent in patients and control subjects, featuring the allele frequencies as a potential ethnic or geographical risk factor. By pooling all studies per continent, we performed the analyses of the dominant models and the genotypes using the fixed-effect model. Our overall results showed no association of the R72P polymorphism with breast cancer, but Asian patients had an increased risk associated with the dominant model RR + RP *vs.* PP (OR = 1.23; 95% CI 1.07-1.41), as described in Table 2. These remarkable data concerning RR + RP genotypes and R allele in Asia are in agreement with the reports that R variant increased breast risk in patients from China (Weston & Godbold 1997; Li et al. 2002) and India (Gochhait et al. 2007). In contrast, previous meta-analysis designed studies failed to correlate the R72P polymorphism with breast cancer (Ma et al. 2006; Zhuo et al. 2009), even when subjects were stratified by ethnicity or source of controls (Ma et al. 2011).

**Table 2:** **Meta-analysis of the R72P polymorphism of the gene** ***TP53*** **on breast cancer, by pooling data per continent**

	RR ***vs.*** RP	RR ***vs.*** PP	RP ***vs.*** PP	RR+RP ***vs.*** PP	RR ***vs.*** RP+PP
	OR (95% CI)	OR (95% CI)	OR (95% CI)	OR (95% CI)	OR (95% CI)
Africa	1.41 (0.90-2.22)	1.30 (0.69-2.44)	0.99 (0.54-1.83)	1.05 (0.6-1.86)	1.32 (0.86-2.01)
America	1.04 (0.87-1.23)	1.04 (0.82-1.33)	1.01 (0.88-1.16)	0.98 (0.86-1.11)	0.96 (0.90-1.03)
Asia	1.05 (0.88-1.26)	1.24 (0.87-1.78)	1.15 (0.85-1.57)	*1.23 (1.07-1.41)	1.04 (0.93-1.17)
Europe	1.09 (0.97-1.22)	1.10 (0.93-1.31)	1.01 (0.92-1.11)	1.04 (0.95-1.13)	1.03 (0.98-1.08)
**Overall**	1.01 (0.97-1.04)	1.05 (0.99-1.12)	1.05 (0.99-1.12)	1.06 (0.99-1.12)	1.05 (0.98-1.05)
**Heterogeneity**	χ^2^ = 3.91	χ^2^ = 5.42	χ^2^ = 5.78	χ^2^ = 6.15	χ^2^ = 4.53
	*P* = 0.27	*P* = 0.14	*P* = 0.12	*P* = 0.10	*P* = 0.21

In conclusion, we found significant association between the R72P polymorphism in the *TP53* gene and the breast cancer risk. The overall data showed an increased risk due to the P allele dominant model, but not in Asia where the risk was associated with the R allele and R dominant model. The present meta-analysis is supported by a large sample size composed by 25,629 cases and 26,633 controls from 41 studies.
